# Histological and histomorphometric study using an ultrasonic crestal sinus grafting procedure. A multicenter case study

**DOI:** 10.4317/medoral.20994

**Published:** 2016-03-06

**Authors:** Marcel Wainwright, Daniel Torres-Lagares, Beatriz Pérez-Dorao, María-Angeles Serrera-Figallo, José-Luis Gutierrez-Perez, Angelo Troedhan, Andreas Kurrek

**Affiliations:** 1 Professor of Master in Oral Surgery, University of Seville, Dentalspecialists Kaiserswerth, Germany; 2 Master in Oral Surgery – School of Dentistry – University of Seville, Spain; 3 Vis. Prof. University of Ventiane, Faculty of Dentistry, Head of Center of Facial Esthetics Vienna, Austria; 4 Vis. Prof. of University of Ventiane, Faculty of Dentistry, Private Practice Dusseldorf, Germany

## Abstract

**Background:**

The aim of this study was to evaluate the efficacy of a hydrodynamic ultrasonic driven transcrestal sinus grafting procedure (Intralift ®, Acteon Company, Bordeaux, France) and the use of a bovine high temperature sintered grafting material in sinus sites with less than 5 mm remaining bone height with no additional autogenous bone in order to create a sufficient recipient site for implants.

**Material and Methods:**

12 patients (16 sinus) in this multicenter case study were included. Using a crestal approach, bone under the sinus was prepared with ultrasonic tips until the Schneiderian membrane was reached. With a trumpet shaped instrument, the Schneiderian membrane was elevated. In the new created subantral space a high temperature sintered bovine grafting material was introduced (Bego Oss, BEGO Implant Systems GmbH & Co. KG, Bremen, Germany). After 6 months biopsies were taken with a trephine bur and histologies were generated following histomorphometric analysis.

**Results:**

The results showed new vital bone in average of 33.4% ± 17.05%, and 43.6% ± 16.70 of bone substitute material. No signs of abnormal inflammation were observed.

**Conclusions:**

This procedure (Intralift ®) allows, using a bovine material with no additional autogenous bone, new bone formation in the sinus in order to allow place implant subantraly.

**Key words:**Bone regeneration, sinus, intralift ®, xenogenic bone graft

## Introduction

One of the keys of success in dental implant treatment is to possess a suitable amount of alveolar bone in the region on which we decide to place the implants as a result of a thorough diagnosis and surgery planning. The posterior area of the maxillary bone has challenging anatomical features mainly due to the presence of the maxillary sinus (1). Extended sinus volume due to progressing pneumatization in areas with missing teeth and additional crestal bone resorbtion as a consequence of bone atrophy cause the need of augmentation (2).

Therefore, to solve the loss of bone height, sinus lift techniques have been developed (Boyne and James) (3), increasing the availability of bone in the posterior maxilla in order to succeed in dental implant treatment. In fact, sinus floor elevation is one of the most frequently performed regenerative procedures in dental implantology. Based on the principle of elevation of the basal sinus mucosa, it enables the insertion of a matrix, promoting bone regeneration in the subsequent healing process (4,5) Tatum (5) subsequently designed a sinus lift via a lateral approach with osteotomy of the vestibular cortical bone, as the space gained after raising the membrane was occupied by a filling material that would maintain a virtual space during the process of bone regeneration. In terms of bone graft materials, autogenous bone unreservedly represented the gold standard a few years ago. Nevertheless, in the last years good results have also been achieved by bone substitute materials, particularly when the height of the residual bone is moderately or extensively reduced. Whereas only an autogenous bone graft contains vital bone cells enabling direct osteogenesis (6-8) autogenous bone is not only limited but also must be obtained from a further operation site, with the disadvantages that it entails of the patient´s morbidity (9,10). Various bone substitute materials have been available for many years; these can be classified according to different aspects. A widely used classification is according to their origin, which distinguishes natural bone matrix from synthetically produced materials. Among the natural materials, human (allogenic) and animal (xenogenic) bone minerals are established. Plant (phycogentic) tissues play a special role. From the chemical aspect, the natural materials consist predominantly of hydroxyapatite. Tricalcium phosphate, synthetically produced hydroxyapatite and the bioactive glasses are synthetically manufactured materials. The beta-form of tricalcium phosphate has demonstrated complete degradability (11). However, the biodegradation was also associated with a volume loss, even though regeneration hardly differs from that of autogenous material (8,12) In theory, the body can react to a non-vital, ceramic bone augmentation material in four different ways: conversion to vital bone tissue with complete absorption of the bone substitute material is called osseous organization. This contrasts with the formation of ceramic-osseous regenerated tissue, in which the substitute is first unsheathed in bone and is absorbed very slowly in the course of natural remodeling. Depending on the absorption kinetics, some bone substitute materials can still therefore be detected histologically even after many years. Usually they appear blandly embedded in the newly produced hard substance. A fibrous sheath around the material in the form of a foreign body reaction must be regarded as a failure, as is infection with subsequent loss of the augmentation. Which of these four possibilities actually takes place after the insertion of a bone graft; it depends on the choice of material employed, along with a number of other factors (13). It should also be noted that all the aforementioned materials have only an osteoconductive effect, unless they are combined with growth factors, so that they only act as a scaffold for bone formation (14,15).

Moreover, the aim of a less invasive technique leads to the use of compressive osteotomes (Summers) to lift the sinus membrane with a closed technique using a crestal approach (16,17), and additional filling of the sinus with different graft materials. Soltan and Smiler (18) proposed a balloon technique (Antral Membrane Balloon Elevation, AMBE), consisting in gently detaching the membrane using a latex balloon inflated with saline solution. This technique offers advantages such as reduced postoperative pain, bleeding and wound infection rates.

Consequently, an important contribution to oral surgery was the introduction of piezoelectric surgery. Thus, Torrella *et al.* (19) proposed the use of piezoelectric surgery for lateral osteotomies. Performed with a bone preserving incision, they are less traumatic and reduce the risk of perforation of the Schneiderian membrane, achieving a better view during surgery. Based on the use of piezoelectric surgery attempts have been made to simplify the sinus lift technique to offer patients an intervention as atraumatic as possible, with milder postoperative discomfort. To this end, Troedhan, Kurrek and Wainwright (20) in conjunction with the Acteon Group (Bordeaux, France) developed the Intralift™, a minimal invasive hydrodynamic elevation technique for lifting the maxillary sinus membrane from a crestal approach, using piezoelectric surgery based on a specific set of tips for the application of ultrasound. This technique opens a wide range of possibilities in terms of reducing the complexity and morbidity of open sinus lift (21-23).

The aim of the present study was introduce the clinical and histological evaluation of BEGO OSS, a natural xenogeneic bone substitute material (BEGO Implant Systems GmbH & Co. KG, Bremen, Germany) for the indications of sinus floor elevation with the Intralift™ technique in atrophic sinus sites with less than 5 mm remaining bone height. The hypothesis was to evaluate, if even in extended atrophic sinus sites augmentation via the Intralift™ technique bone formation with a bovine material without adding autologenous bone was efficient for bone formation and secondary implant placement compared to immediate implant with simultaneous sinus grafting.

## Material and Methods

Twelve healthy patients were included in the study (n = 12; average age 50.5 years, range 31 to 79 years). All patients had good oral hygiene but had at least one free-end or gap situation in the posterior maxilla requiring implant rehabilitation. The boundary between a one-stage and two-stage sinus lift was set at a residual bone height of 5 mm, based on the expected primary stability of the implants that would be inserted. Some of the procedures included augmentation of other regions. Nine simultaneous and six two-stage sinus lifts took place in total. All patients included had no reaction or pathologies in the sinus mucosa on radiographic analysis via CBST. All ethical comittee´s criteria were aligned and fulfilled: the patients fulfilled informed consent, and protocol of study was approved by Ethical Committee of University of Seville.

- Surgical procedure

Under local anesthesia, a crestal incision was made, a mucoperiosteal flap was raised and from a crestal osteotomy using ultrasonic tips the sinus floor was entered and the Schneiderian membrane was exposed. The Intralift™ procedure is based on 5 diamond coated, laser marked tips increasing in width to perform a crestal osteotomy to reach the sinus floor with its onlaying membrane. The last but one tip (TKW4) has a diameter of only 2.8 mm and allows the trumpet shaped tip (TKW5) to enter. In areas with extended sinus two crestal osteotomies were performed. The crestal sinus floor was fenestrated with piezosurgery and the Schneiderian membrane was cranialized, using a trumpet shaped tip with a central orifice that created a cavitation effect with high frequency activated irrigation (TKW5). The piezocrystal in the handpiece is creating a high frequency between 20-25 kHz that causes energy, which is transferred into ultrasound. In presence of a liquid (i.e. saline, Ringer, ozonized water) and if the pressure is exceeding 10W/cm2, micro bubbles are imploding immediately and release kinetic energy to detach the Schneiderian membrane from the sinus floor. In the two-stage procedure, the bone substitute BEGO OSS with a particle size of 1-2 mm was then introduced with a volume of 1-5 ml. This phenomenon is described as the cavitation effect and the elevation of the Schneiderian membrane follows due to the physics of the cavitation effect a circumferential detachment that allows creating a new subantral space for placement of bone substitute material. Adding of any autogenous bone was omitted. Prior to the filling with the bovine material, collagenous sponges were introduced into the osteotomy (Gelastypt, Curasan Germany) to prevail micro ruptures caused by the geometry of the bone substitute particles and avoid iatrogenic perforation of the Schneiderian membrane whilst the use of the trumpet (TKW5). The trumpet shaped instrument (TKW5) worked as a plugging instrument to fill the subantral space with the augmentation material. In the single-stage procedure, one or more implants (BEGO Semados, S-Line, BEGO Implant Systems GmbH & Co. KG, Bremen, Germany) were placed at the same time according to the respective manufacturer’s instructions. Finally, the crestal sinus osteotomy was covered with a native pericardial membrane (BEGO collagen membrane, BEGO Implant Systems GmbH & Co. KG, Bremen, Germany) and the soft tissue was closed by suture. The treatment concluded with a postoperative radiograph.

**Figure 1:**
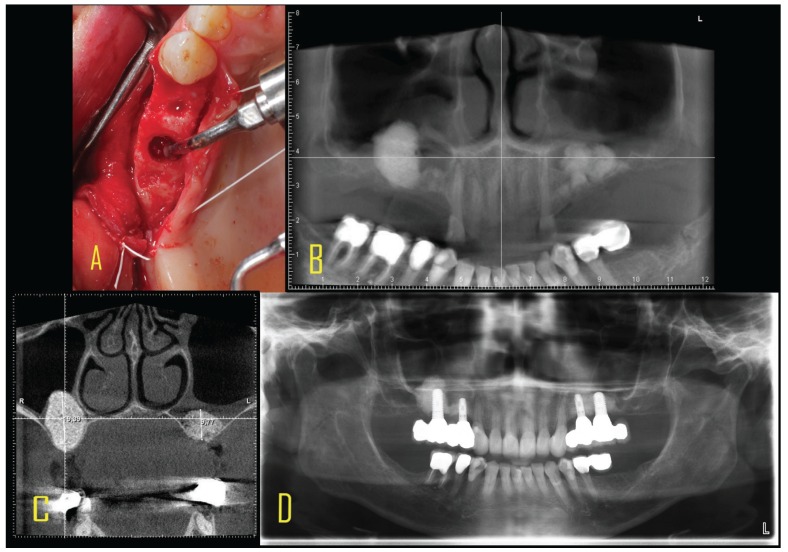
A: Intraoperative site with using the TKW5 trumpet as a plugging instrument for the bone grafting material into the sinus. B: CBST post op of bilateral hydrodynamic ultrasonic based elevation and augmentation with bovine grafting material (BEGO Oss. BEGO Germany). C: Cross-sectional view of both augmented sites. residual bone height was less than 1.5 mm on the right and left maxilla. Height gain was achieved in the right subantral space up to 19.39 mm and on the left site up to 9.77 mm. D: Panorex one year after implant loading with fully osseointegration and stable periimplant bone conditions.

Follow-up usually took place one and 7-10 days postoperatively and six months later at the exposure operation or implant placement with a further radiograph. In the two-stage procedure, with the patients’ consent, the primary drilling for positioning an implant was replaced by trephine drilling. This was followed by further implant insertion according to the respective manufacturer’s instructions and a radiograph was taken (Fig. 1). In the one-stage procedure cases, a biopsy was taken near implants, in a zone with sinus elevated six months ago.

- Histological procedure

**Figure 2:**
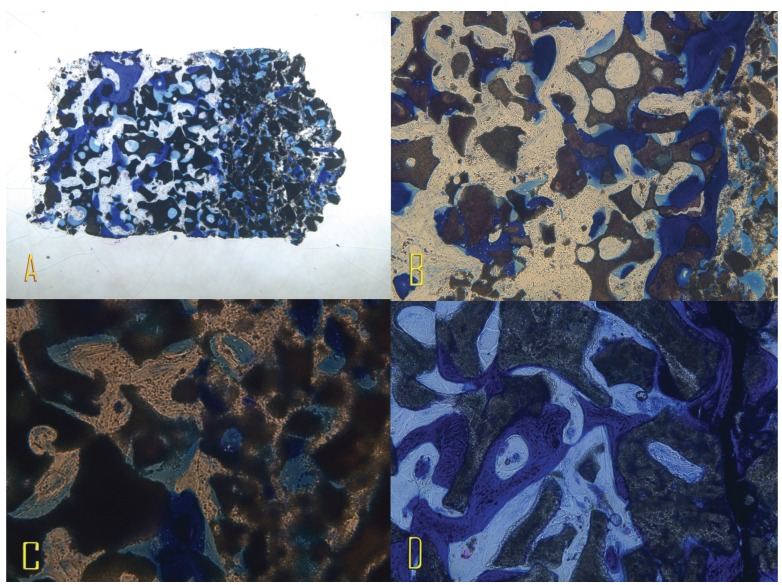
A: 4x magnification Toluidin blue stained trephine sample. B: 100x magnification Toluidin blue stained trephine sample. C: 200x magnification Toluidin blue stained trephine sample. D: 400x magnification Toluidin blue stained trephine sample.

The biopsies were fixed in 4 % formalin solution and processed for hard tissue histology using the Donath semi thinground technique. After toluidine blue staining they were assessed histologically using a light microscope (Olympus BX50, Olympus Iberia SAU, Barcelona, Spain) with the aid of a CCD camera. In addition to the general histological assessment, the augmented regions were defined as the “region of interest” using a computer-based program (Cell D, Soft Imaging System, Muenster) and the area of newly formed bone and residual bone substitute material it contained were determined (Fig. 2). All variable data was introduced in mean and standard deviation.

## Results

Healing was uneventful in all patients. There was no suture dehiscence, prolonged swelling or wound infection. The postoperative radiography after exposure and prior to implant insertion showed clearly stable augmentation volume in all cases. The bone of the crestal osteotomy had regenerated clinically in all cases, while isolated granules could still be faintly distinguished from the surrounding hard tissue. New hard tissue had formed between and on the individual particles, which felt firm and similar to bone on palpation. No dislocation or absorption of the augmented material was found. In the two-stage procedures, all implants achieved adequate primary stability. The histology of all trephine biopsies showed complete bony regeneration of the augmentation (Fig. 2). The bone substitute granules (gray) could be distinguished clearly from the newly formed bone (blue) because of their different staining behavior. The majority of the individual bone substitute particles were surrounded by a thin bone matrix and were also seen to be linked by newly formed bone trabeculae. Normal bone marrow was visible between the hard tissue. Absorption of the material was not observed after a maximum healing period of six months. Figures 2 show photographs of the trephine histologies, and the patient data and results of histomorphometric analysis are presented in  Table 1. The results showed new vital bone in average of 33.4% ± 17.05%, and 43.6% ± 16.70 of bone substitute material. No signs of abnormal inflammation were observed.

**Table 1:**
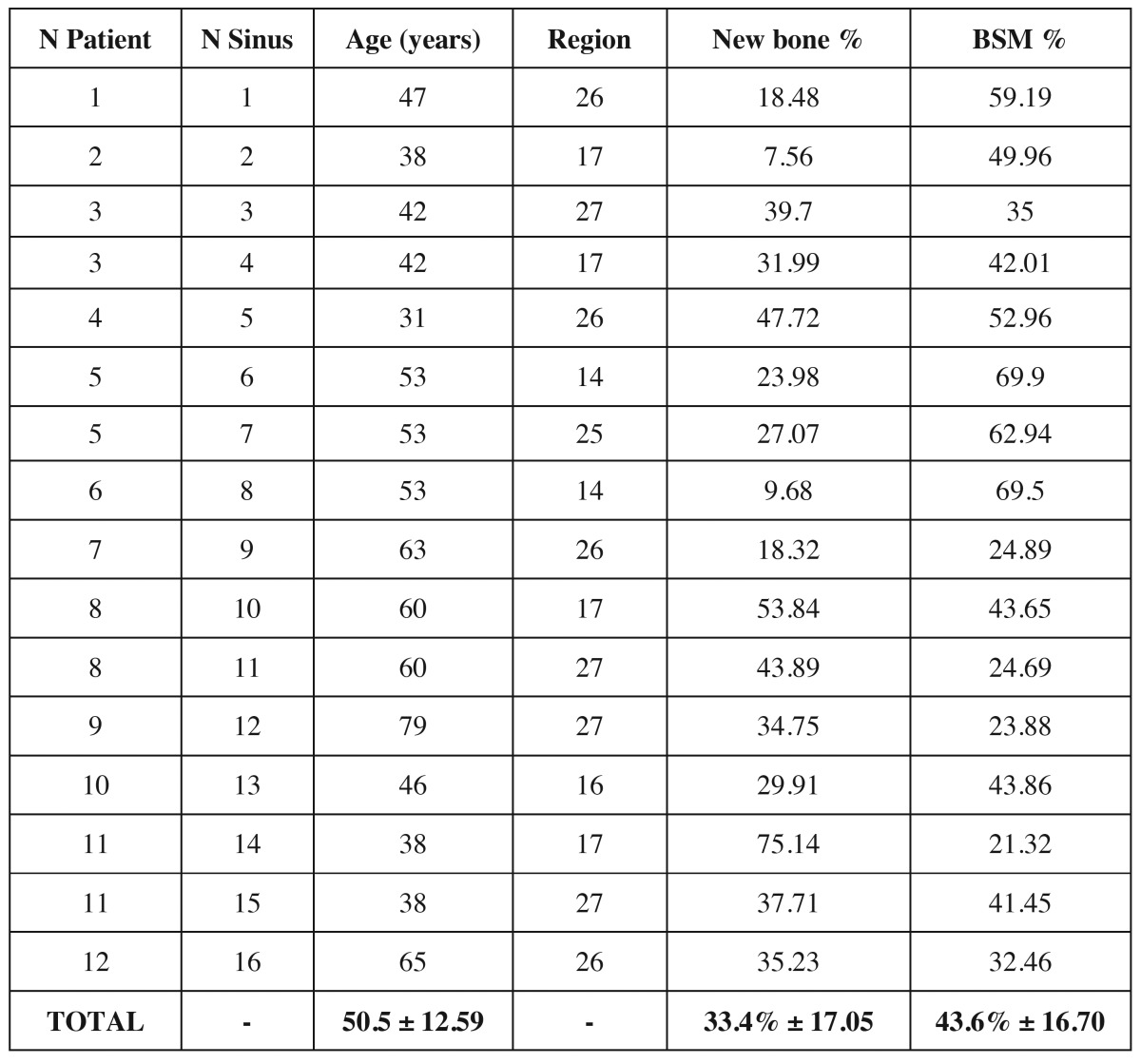
Result of the biopsies (BSM: Bone Substitute Material).

## Discussion

In the present study, the suitability of a non-absorbable granular bone substitute material for sinus floor elevation was evaluated in conjunction with the Intralift technique, a minimal invasive crestal ultrasonic driven sinus grafting procedure. After a healing period of six months, good bony consolidation of the augmentation was seen clinically and histologically in all patients. If the current literature is considered, a number of studies can be found on the most varied augmentation materials in sinus floor elevation. The xenogeneic natural bone minerals exhibit predictably good results. A recent review article even questions autogenous bone alone as “gold standard” for sinus floor elevation since complete regeneration can be achieved precisely in the sinus with bone substitute materials alone (9). In a direct comparison of xenogeneic and alloplastic bone substitute materials, a mixture of autogenous bone and ß-TCP (1:1) demonstrated a significantly lower proportion (32 %) of newly formed bone histologically after a twelve-month healing period than a mixture of autogenous bone with xenogeneic bone mineral (46 %). The authors attributed this to a better guide function of the xenogeneic material (2). In this study, the observed area of newly formed bone was 7.56-75.14 %. It should be taken into account that the augmentations were performed without the admixture of autogenous material and thus without further patient morbidity due to the associated autogenous bone harvesting. In this connection, a review of 5128 implants, which were evaluated up to 102 months after insertion, found a better long-term implant survival rate after external sinus floor elevation with the use of xenogeneic granules alone than with autogenous or alloplastic material (1). The histology of the biopsies obtained in the present study indicates that the material possesses good osteoconductivity (Fig. 2). The histological appearance is similar to other xenogeneic materials (20) if the lateral sinus wall is covered with a collagen membrane. In this study, the granules of a different xenogeneic substitute material were also connected through newly formed bone trabeculae after healing for six months and did not show any inflammatory reaction in the stroma.

In the present study, admixture of autogenous bone was omitted. For this reason, regeneration could originate only from the basal and lateral parts of the osseous sinus or the Schneiderian membrane (24). Covering the osteotomy with a collagen membrane in the sense of guided bone regeneration makes sense in order to prevent soft tissue from growing into the space of the bone defect (5). Moreover, sinus floor elevation is readily combined with other augmentation procedures in which periosteal splitting may be necessary, causing further exposure of soft tissue. In the literature, covering the (lateral) window led to a significant increase in the rate of implant survival (7,20).

The bone substitute used in this study differs from other xenogeneic natural bone minerals (e. g. BioOss, Geistlich Biomaterials, Switzerland) especially as it is sintered by high-temperature treatment. In addition, the surface is roughened by acid treatment. The use of high temperatures is intended to ensure a further degree of safety while the acid treatment increases the hydrophilia of the surface. On the other hand, sintering at high temperatures results in an increase in absorption stability. It has been shown for other xenogeneic materials that some of the bone substitute material can be detected histologically ten years after augmentation even without a sintering process (15). The extent to which the bone substitute material used in this study is incorporated in the bone’s natural remodeling and thus is converted into vital bone in the long term can only be established by further long-term histological studies.

The good histological and clinical results of xenogeneic bone substitute materials can be attributed to a number of factors. On the one hand, these materials are highly biocompatible on account of their biological origin and have similar biochemical properties to those of human cancellous bone. In addition, their pore size resembles that of human bone. It was shown for synthetic hydroxyapatite that pore sizes below 100 μm can prevent the ingrowth of blood vessels and thus new bone formation (8,11). All of this may support the good osteoconductivity and thus rapid regeneration of xenogeneic augmentations. Moreover, clearly improved volume stability is ensured because of the slow absorption, compared with autogenous bone (17).

Conclusion

The autogenous bone in Sinus grafting procedures could not be longer considered still the gold standard. It was concluded that the integration of the investigated bone substitute material BEGO OSS (BEGO Implant Systems GmbH & Co. KG, Bremen, Germany) into bone can be predicted after it is used for sinus floor elevation, even if there is no autogenous bone added and the bone substitute material is used alone. Within the follow-up period of six months, there is no significant absorption and volume stability is good. Direct comparison with other bone substitute materials requires further clinical studies.
